# Supplementary material for: Identification of photoperiod-induced specific miRNAs in the adrenal glands of Sunite sheep (Ovis aries)
Source: Front Vet Sci. 2022 Jul 22;9:888207. doi: 10.3389/fvets.2022.888207 (PMC9354845; doi:10.3389/fvets.2022.888207)
Supplement: Supplementary file 1 [file Data_Sheet_1.ZIP › Supplementary file/miRDeep2 file.zip!/miRDeep2 file/Know_Structure/LP42A3/oar-mir-221.pdf]

miRBase precursor	: oar-mir-221
Total read count	: 2601
oar-miR-221 read count	: 2347
remaining reads	: 254

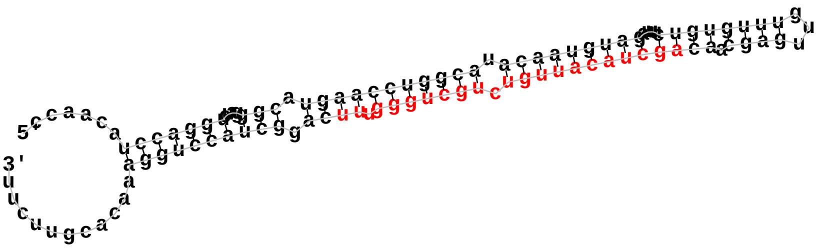

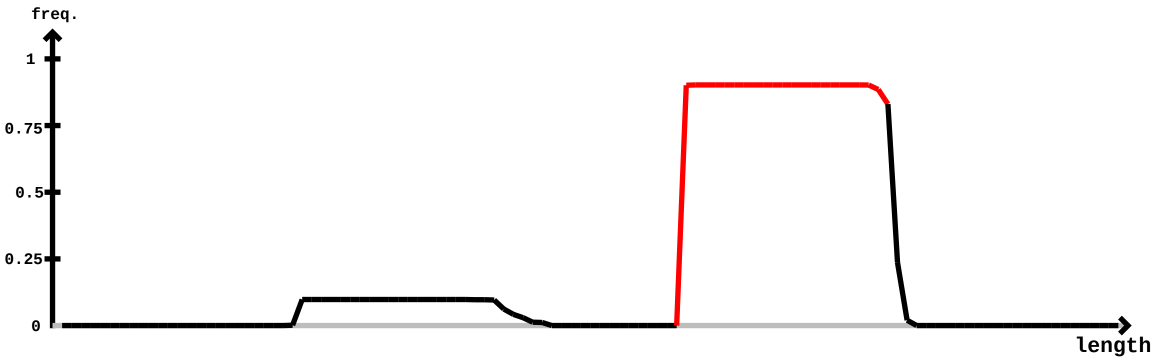

oar-miR-221

```
5'- ccaacauccaggucuagggcaugaaccuggcauacaauguagauuucuguguuuguugagcaacagcuacauugucugcuggguuucaggcuaccuggaaacacguucuu   -3'    exp
    ......(((((((....(((.(((((((((((.(((((((((....((((((((...)))).))))))))))))).))))))).)))).))))))))))...........    reads      mm         sample
    .......................aaccuggcauacaauguagauu.................................................................    2          0          seq
    .......................aaccuggcauacaauguagauuu................................................................    1          0          seq
    ........................accuggcauacaauguag....................................................................    2          0          seq
    ........................accuggcauacaauguagau..................................................................    3          0          seq
    ........................accuggcauacaauguagauu.................................................................    86         0          seq
    ........................accuggcauacaauguagauuu................................................................    51         0          seq
    ........................accuggcauacaauguagauuuc...............................................................    32         0          seq
    ........................accuggcauacaauguagauuucu..............................................................    44         0          seq
    ........................accuggcauacaauguagauuucug.............................................................    3          0          seq
    ........................accuggcauacaauguagauuucugu............................................................    30         0          seq
    ................................................................agcuacauugucugcuggg...........................    1          0          seq
    ................................................................agcuacauugucugcugggu..........................    44         0          seq
    ................................................................agcuacauugucugcuggguu.........................    142        0          seq
    ................................................................agcuacauugucugcuggguuu........................    1543       0          seq
    ................................................................agcuacauugucugcuggguuuc.......................    562        0          seq
    ................................................................agcuacauugucugcuggguuuca......................    52         0          seq
    .................................................................gcuacauugucugcuggguuu........................    2          0          seq
    .................................................................gcuacauugucugcuggguuuc.......................    1          0          seq
```
